# Error in Figure

**DOI:** 10.1001/jamanetworkopen.2026.38355

**Published:** 2026-09-14

In the Original Investigation titled “Vision Screening and Vision Loss–Related Conditions Among American Indian or Alaska Native Children,”^1^ published July 13, 2026, there was an error in Figure 3. The originally published Figure showed only the overall vision screening rates among the 2 insurance types (public or Medicaid vs private or employer based), rather than age-specific vision screening rates by insurance types. This article has been corrected.^1^
